# The Incorporation of Plant-Derived Polysaccharides into Alginate-Based Capsules Improve Probiotic Viabilities During Storage, Gastrointestinal Digestion, and Their Application in Yogurt

**DOI:** 10.3390/foods15010163

**Published:** 2026-01-03

**Authors:** Sijia You, Xinming Zhao, Weina Cui, Huan Liu, Jielun Hu

**Affiliations:** Key Laboratory of Bioactive Polysaccharides of Jiangxi Province, China-Canada Joint Laboratory of Food Science and Technology (Nanchang), State Key Laboratory of Food Science and Resources, Nanchang University, Nanchang 330047, China; yousijia@email.ncu.edu.cn (S.Y.); zxm18482104090@163.com (X.Z.); 357900230038@email.ncu.edu.cn (W.C.)

**Keywords:** probiotic, microencapsulation, polysaccharides, alginate, yogurt

## Abstract

The objective of this research was to combine three plant-derived polysaccharides, *Lycium barbarum* polysaccharides (LBP), peach gum polysaccharide (PGP), and citrus pectin (CP), with alginate (SA) to co-encapsulate probiotics and investigate the survival of cells during in vitro gastrointestinal digestion, storage, and application in yogurt. The incorporation of different polysaccharides into SA all improved the encapsulation efficiencies and surface regularities of probiotic capsules. Texture analysis showed that the PGP-incorporated microspheres exhibited the highest values for hardness, springiness, and resilience, while in terms of chewiness, the highest values were observed for the LBP and CP groups. In vitro gastrointestinal digestion analysis revealed that the incorporation of different polysaccharides all further enhanced the cell survival rates, and the SA: PGP group demonstrated superior probiotic protection with the minimal viability loss of only 0.40 log CFU/g after 6h digestion. During storage, SA: PGP group also exhibited the highest stability, which still maintained 7.7 Log CFU/g of viable cells at the end of 20 days storage, and after incorporation of SA: PGP into yogurt, 7.8 Log CFU/g of viable cells were still detected at the end of 21 days storage.

## 1. Introduction

As defined by WHO/FAO, probiotics are live microorganisms that, when administered in adequate amounts, confer a health benefit on the host [1]. Critical colonization threshold (>6 log CFU/g in the gut) enables probiotics to exert multifaceted physiological effects [2], including: enhancement of immune responses, maintenance of intestinal homeostasis [3], mitigation of inflammatory bowel disease (IBD) [4], and alleviation of diarrheal [5,6]. However, probiotics exhibit high sensitivity to various environmental stressors, including thermal processing, oxygen exposure, water activity fluctuations, pH variations, enzymatic degradation, and bile salt challenge during food manufacturing, storage, and gastrointestinal transit. These factors collectively diminish probiotic viability, consequently impairing their bio-accessibility and intended physiological benefits. To achieve optimal therapeutic efficacy, maintaining adequate viable cell counts (>10^6^ CFU/g) in final products prior to consumption represents a critical technological requirement [7]. Consequently, effective strategies, such as microencapsulation technology, are needed to improve the stability of probiotics in foods and during their passage through the human gut.

Among the wall materials applied for microencapsulation, sodium alginate (SA) has been widely selected in food and pharmaceutical applications for probiotic controlled release [8], which owned the advantage of mild processing conditions, simple operation, and excellent biocompatibility [9,10,11]. However, Ca^2+^-crosslinked alginate hydrogels suffered from inherent limitations: they destabilized in alkaline conditions (pH > 7.0) due to competitive chelation of calcium ions [12], and the highly porous structure led to low encapsulation efficiency, rapid payload leakage, and inadequate protection against oxygen, acids, and digestive enzymes [13]. To address these shortcomings, a composite encapsulation strategy was accordingly developed to enhance both the microstructural density and gastrointestinal stability of sodium alginate-based gel systems. However, the types of wall materials commonly combined with SA for enhanced probiotic encapsulation are still limited and thus need further exploration.

Polysaccharides are natural polymers formed by the condensation of 10 or more monosaccharide units linked together by glycosidic bonds [14]. As essential biomacromolecules in living organisms, they were renowned for the biocompatibility, biosafety, and various biological activities such as exhibiting immunomodulatory and anti-inflammatory properties; demonstrating antioxidant, anti-tumor, lipid-lowering, and hypoglycemic capabilities [15,16,17,18]. Currently, several polysaccharides have been employed in combination with SA to encapsulate probiotics, leading to enhanced protective effects. As demonstrated by Wei et al., a sodium alginate/pectin composite was used to encapsulate *Lactobacillus plantarum*, and found that the addition of pectin not only preserved high encapsulation efficiency but also markedly improved the acid resistance and enteric release profile of the probiotic [19]. Ming et al. constructed capsules using sanxan gum combined with SA to deliver/protect *L. plantarum*, and found that capsules with the addition of sanxan gum significantly enhanced the resistance of probiotics to the acidic components and digestive enzymes in gastric acid fluid, as compared with the single alginate-based capsules [20]. Although microencapsulation using SA-polysaccharide complexes has demonstrated synergistically enhanced protection for probiotics, the variety of polysaccharides developed for this purpose remains limited, thus creating a pressing need to diversify available options.

In this study, three plant derived polysaccharides, *Lycium barbarum* polysaccharides (LBP), peach gum polysaccharide (PGP) and citrus pectin (CP), were applied to co-encapsulate probiotics with SA to firstly, characterize the encapsulation efficiency, morphology, texture and rheological properties of probiotic capsules; secondly, the in vitro gastrointestinal digestion and storage stability properties were carried out; finally, the fabricated probiotic capsules were applied in yogurt and the corresponding volatile flavor profile and storage stability were characterized.

## 2. Materials and Methods

### 2.1. Materials

*Lacticaseibacillus rhamnosus* (Guangdong Microbial Culture Collection Center), *Lycium barbarum* polysaccharides (Evergreen Bio, Xi’an, Shaanxi Province, China, Mw ~65 kDa, carboxyl content ~4.18 ± 0.21 mmol/g), peach gum polysaccharide (Shanghai Yuanye Biotechnology Co., Ltd., Shanghai, China, Mw ~380 kDa, carboxyl content ~1.05 ± 0.08 mmol/g), citrus pectin (Jiangsu Ruiduo Biotechnology Co., Ltd., Nanjing, Jiangsu Province, China, Mw ~34 kDa, carboxyl content ~12.35 ± 0.42 mmol/g, DE = 38.6 ± 1.2%), sodium alginate (SA) were purchased from Aladdin Biotechnology Co., Ltd., Shanghai, China. Pepsin and trypsin were purchased from Soleibao Co., Ltd., Beijing, China. Other related reagents, such as calcium chloride, are analytical pure grade and purchased from Xilong Science Co., Ltd., Shantou, Guangdong Province, China. All the solutions were prepared with distilled water.

### 2.2. Preparation of Probiotic Capsules

Encapsulation was performed utilizing whey protein isolate (WPI) thermogelation and Ca^2+^-induced “egg-box” crosslinking of sodium alginate (SA) [21,22]. Wall material solutions were prepared at a constant total solid concentration of 9% (*w*/*v*) as follows: 2% (*w*/*v*) SA solution and 2% (*w*/*v*) plant-derived polysaccharide (LBP/PGP/CP) solutions were separately dissolved in distilled water under magnetic stirring (RHS025, IKA, GmbH & Co. KG, Staufen, Baden-Württemberg, Germany) at 25 ± 1 °C for 4 h to achieve complete dissolution and hydration. The two solutions were then mixed at volume ratios of 1:2, 1:1, and 2:1 (SA: polysaccharides) with continuous magnetic stirring for 30 min to ensure homogeneous mixing. Subsequently, 60 mL of each SA-polysaccharide solution was homogenized with 40 mL of heat-denatured 5% (*w*/*v*) WPI solution (pre-heated at 85 °C for 15 min and cooled to 25 °C) using a cantilevered agitator (RW 20, IKA, Germany) at 5000 rpm for 20 min under constant temperature control (25 ± 1 °C). The washed *Lacticaseibacillus rhamnosus* (LGG) suspension (1 × 10^10^ CFU/mL) was aseptically incorporated into 100 mL of the wall material mixture at a volume ratio of 5% (*v*/*v*) under continuous magnetic stirring (600 rpm) for 15 min to ensure uniform dispersion of probiotics. The resulting core-wall mixture was extruded via an automatic syringe pump (T3, Shandong Zibo Guanjie Electronic Technology Co., Ltd., Zibo, Shandong Province, China) equipped with a 0.8 mm inner diameter needle, which was vertically fixed 5 cm above the coagulation bath surface. The extrusion rate was precisely controlled at 1 mL/min to deliver the mixture into a 1% (*w*/*v*) CaCl_2_ coagulation bath (pre-adjusted to pH 6.5 ± 0.1 and maintained at 25 ± 1 °C), where capsules formed and underwent ionic cross-linking for 10 min. Following solidification, the capsules were collected by filtration through sterile gauze, washed thoroughly with three aliquots (10 mL each) of sterile distilled water to remove unreacted CaCl_2_, gently blotted dry with sterile filter paper, and stored at 4 °C for further use [23,24].

### 2.3. Determination of Encapsulation Efficiency (EE)

Capsules (1 g) were decapsulated in 9 mL of 0.2 M PBS using a high-speed disperser (4000 rpm, 10 min) (T-18, IKA, GmbH & Co. KG, Staufen, Baden-Württemberg, Germany). The released suspension was serially diluted in 0.2 M PBS, plated on MRS agar. The 37 °C, 48 h incubation was followed by colony enumeration and *EE* calculation:

*EE = N/N*_0_ × 100%

where:

*EE* = Encapsulation efficiency (%)

*N* = Viable count released from capsules (CFU/g)

*N*_0_ = Initial viable count (CFU/g)

### 2.4. Characterization of Probiotic Microspheres

#### 2.4.1. Particle Size Measurement

Ten capsules from each experimental group were randomly selected. The diameter of individual capsules was measured using a digital micrometer (DL321025S, Deli Group Co., Ltd., Ningbo, Zhejiang Province, China) with an accuracy of 1 μm. For each capsule, three independent measurements were taken along mutually perpendicular axial orientations (i.e., length, width, and height) to minimize dimensional bias caused by non-ideal sphericity, and the mean particle diameter was calculated accordingly. Data presented as mean diameter ± standard deviation.

#### 2.4.2. Stereomicroscopy Observation

The morphological characteristics of the capsules were examined using stereomicroscopy (SZX10, Olympus Corporation, Tokyo, Japan). Samples were uniformly dispersed in ultrapure water and observed under an optical stereomicroscope at 10× magnification on the clean glass slides. Digital images were captured using a camera system to evaluate surface morphology, structural integrity, and size distribution.

#### 2.4.3. Texture Profile Analysis (TPA)

Texture analysis was performed using a TA-XT plus texture analyzer (SMS UK Ltd., Godalming, Surrey, UK) equipped with a 36 mm cylinder probe, modified from Huang et al. Parameters were set to pre- and post-test speeds of 0.5 mm/s and a trigger force of 5 g. Capsules were compressed twice (40% strain, 0.1 mm/s). Hardness, elasticity, cohesiveness, chewiness, and resilience were determined from five replicates at 25 °C.

#### 2.4.4. Rheological Characterization

Steady shear measurements were conducted on a TA ARES-G2 rheometer (SMS UK Ltd., Godalming, Surrey, UK) equipped with 25 mm parallel plates, following a protocol adapted from Huang et al. [25]. The linear viscoelastic region (LVR) was identified by performing a strain sweep from 0.00126% to 10% at a frequency of 10 rad/s, and a 0.01% frequency sweep from 0.1–10 Hz (within the LVR). Storage modulus (G′) and loss modulus (G″) were determined, with quadruplicate measurements conducted at 25 °C.

### 2.5. Simulated Gastrointestinal Digestion

The in vitro gastric digestion was simulated using a constant-temperature shaker (Model THZ-100B, Shanghai Hengke Scientific Instruments Co., Ltd., Shanghai, China). Microspheres (1.0 g) and sterilized artificial gastric fluid (0.32% pepsin, 0.2% NaCl of sterile water, pH adjusted to 2.0 with 1 M HCl solution) were used, which was set to maintain a temperature of 37 °C and an agitation speed of 180 rpm throughout the incubation period. At each time point (60 and 120 min), samples were taken for viable cell counting as described previously. A modified version of the sequential SGF and SIF treatment described by Liu et al. was implemented [26]. At the end of digestion with gastric fluid, 10 mL sterile simulated intestinal fluid (0.8% porcine bile salts and 0.1% trypsin of 0.2 M PBS, pH 7.0) was added, and then again incubated at 37 °C for a further 4 h [27]. Upon completion of each phase, samples were taken for viable cell count analysis. Free LGG was also conducted using identical assay methods.

### 2.6. Preparation of Probiotic Yogurt

Preparation of yoghurt used previous methods with modifications [28]. Pasteurized milk was heated to 85 °C for 20 min under continuous stirring to induce protein denaturation and viscosity development. After cooling to ambient temperature (≤37 °C), 30% (*v*/*v*) starter culture—Junlebao original fermented yogurt (the core strains: Streptococcus thermophilus and Lactobacillus bulgaricus) was inoculated. The mixture was transferred to a 37 °C incubator for 12 h to facilitate fermentation, yielding homemade yogurt. Post-fermentation, 3% (*w*/*v*) probiotic capsules and 5% (*w*/*v*) sucrose were aseptically incorporated into the yogurt under homogeneous mixing.

### 2.7. Yogurt E-Nose Analysis

Volatile profiles of yogurt were captured using a PEN 3 e-nose (Airsense Analytics GmbH, Schwerin, Mecklenburg-Vorpommern). This system utilizes a 10-sensor array of metal oxide sensors, each demonstrating a tailored responsiveness to particular volatile organic compounds.

The PEN-3 E-nose sensor array corresponds to the following volatile compound classes: W1C: Aromatic hydrocarbons (e.g., benzene derivatives); W3C: Ammonia and aromatic compound mixtures; W6S: Hydrogen-containing volatiles; W5C: Nonpolar organics (alkanes, aromatic hydrocarbons); W1S: Short-chain alkanes (e.g., methane); W1W: Sulfur-containing compounds (e.g., H_2_S) and terpenoids; W2S: Alcohols and partial aromatic compounds; W2W: Aromatic compounds and organic sulfides; W3S: Long-chain alkane fractions; W5S: Nitrogen oxide-containing volatiles

The specific operation method of the electronic nose was adapted with minor changes from the procedure described by Zeng et al. [29]. Firstly, yogurt within a probiotic capsule was accurately weighed into the headspace injection bottle using an electronic analytical balance, and then sealed airtight with 3 layers of plastic wrap. The sealed probiotic capsules were incorporated into the yogurt and incubated in a water bath at 40 °C for 20 min, followed by cooling to room temperature for final use. The parameters of the electronic nose were set to a 120 s measurement, a 1 L/min flow rate, and a 150 s cleaning.

### 2.8. Storage Stability

Storage of the capsules was carried out in sterile tubes at a constant 4 °C. At scheduled intervals (days 0, 5, 10, 15, and 20), 1 g samples were collected, homogenized in 9 mL of 0.2 M PBS, and centrifuged (4000 rpm, 10 min). The resulting supernatants were serially diluted, plated on agar, and incubated for colony enumeration. Meanwhile, the storage stability of the probiotic encapsulated yogurt was determined following the established protocol.

### 2.9. Data Analysis

Data were obtained from three independent biological replicates and denoted as mean ± SD. A one-way ANOVA was employed, and subsequent Tukey’s HSD testing was applied to determine differences in specific multiple comparisons, with a *p*-value < 0.05 deemed statistically significant.

## 3. Results and Discussion

### 3.1. Encapsulation Efficiency of Probiotic Capsules

The encapsulation efficiencies (EE) of probiotic capsules were summarized in Table 1. The lowest EE was observed in the group encapsulated with SA alone, reaching approximately 79%. Compared to the control group (SA), the EE of capsules prepared using SA with different polysaccharides (LBP/CP/PGP) improved significantly, which was closely related to their structural characteristics. Their high carboxyl content and high Mw enhanced ionic cross-linking with Ca^2+^ and chain entanglement with SA’s guluronate blocks, forming a dense network that reduced probiotic leakage. Similar results can also be found in Ta et al., who developed sodium alginate-based probiotic capsules and observed significantly higher encapsulation efficiencies supplemented with polysaccharides like κ-carrageenan, locust bean gum, and xanthan gum, as their optimized microcapsule morphology and reduced probiotic leakage [30,31]. In addition, for each sample, with the concentration of SA increased, the EE improved correspondingly, as increased concentration of guluronate (G) residues in SA facilitated stronger coordination with Ca^2+^ ions and formed a denser network that minimized probiotic leakage [32]. Yang et al. fabricated a hybrid gel network via the synergistic interaction between CP and SA, and demonstrated that increasing the concentration of SA resulted in a more densely cross-linked network between SA and CP and thus presented a significant improvement in EE [33]. Notably, among samples with the same ratio, SA: LBP exhibited the highest EE. This was attributed to its abundant hydroxyl groups forming extensive hydrogen bonds with SA’s carboxyl and hydroxyl groups, which complemented the ionic cross-linking of SA-Ca^2+^ and further densified the capsule matrix [34]. The synergistic effect between polysaccharide carboxyl/hydroxyl groups and SA’s G blocks was critical: for LBP, the optimal SA: polysaccharide ratio (2:1) balanced the ionic cross-linking (SA-Ca^2+^) and hydrogen bonding (SA-LBP), maximizing EE. For CP, the high carboxyl density led to saturated cross-linking even at lower SA ratios, resulting in slightly lower EE than LBP.

Among the different capsules prepared, samples with SA to polysaccharide ratio of 2:1 all exhibited the highest encapsulation efficiency and thus were consequently selected for further analysis.

### 3.2. Morphological Characterization of Probiotic Capsules

**Figure 1:**
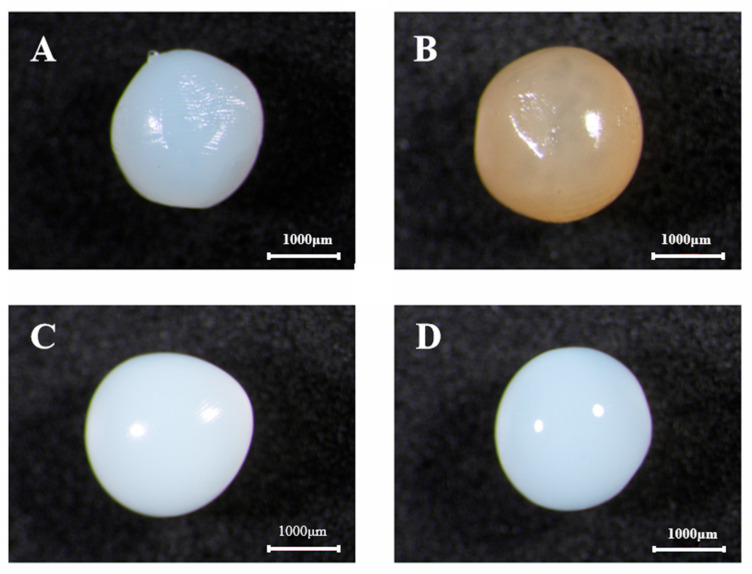
Stereo microscope images of different probiotic capsules. (**A**): SA; (**B**): SA: LBP; (**C**): SA: CP; (**D**): SA: PGP.

Stereomicroscopy was used to characterize the morphology of probiotic capsules. As shown in Figure 1, all capsules maintained spherical morphology with different degrees of sphericity. Specifically, the SA sample exhibited obvious surface wrinkling and internal gas pockets, which can be attributed to the weakened strength of the alginate matrix caused by uneven crosslinking during microsphere formation. SA: LBP and SA: CP microspheres exhibited a moderately smooth morphology with reduced surface glossiness. This structural regularity primarily stemmed from the chain entanglement between guluronate blocks in SA and galacturonate chains in CP, which facilitated a three-dimensional network with enhanced density and stability. As a polyhydroxy compound, LBP possessed abundant hydroxyl groups along its molecular chains, which engaged in extensive hydrogen bonding with the carboxyl and hydroxyl groups on SA, thereby promoting a morphological structure with a higher cross-linking density. Eventually, the SA: PGP sample displayed the most uniform and glossy appearance, which can be attributed to the inherent coiled polymeric chain conformation of PGP that promoted molecular entanglement with alginate and thereby refined the capsule architecture during encapsulation.

The particle sizes of different probiotic capsules were summarized in Table 2. The control sample (SA) exhibited the smallest particle size, measuring 2.14 mm in diameter. In contrast, the incorporation of different polysaccharides resulted in significantly larger capsules, with diameters ranging from 2.25 to 2.37 mm among various formulations. This size enhancement principally stemmed from polysaccharide-induced void filling within the alginate matrix, which increased structural bulkiness and improved sphericity. Among all formulations, the SA: PGP group exhibited the maximum average particle size, reaching 2.37 mm. The observed gradient in particle size directly correlated well with the molecular weight of the polysaccharides (PGP > LBP > CP). Zhang et al. prepared gelatin-tapioca starch microspheres via the water-in-water emulsion technique and found that the incorporation of high-molecular-weight gelatin significantly increased the microsphere diameter compared to low-molecular-weight samples [35].

### 3.3. Texture Analysis of Probiotic Capsules

To evaluate the mechanical properties (hardness, springiness, cohesiveness, chewiness, and resilience) of probiotic capsules, texture profile analysis (TPA) was employed (Table 3). The incorporation of polysaccharides significantly enhanced the hardness, chewiness, and resilience properties of microspheres compared to the SA sample, which can be attributed to the synergistic ionic interactions and chain entanglement between plant-derived polysaccharides and alginate that densified the capsule matrix. Specifically, the PGP-incorporated microspheres [36] exhibited the highest values for hardness, springiness, and resilience. This superior performance can be attributed to the unique helical conformation of PGP, which promoted enhanced physical entanglement with the sodium alginate matrix. This synergistic interaction facilitated more efficient energy storage and release upon deformation, thereby imparting the observed improvements in springiness and resilience. The enhanced hardness after incorporating PGP can be attributed to the synergistic contribution of the filling effect and competitive hydration of PGP within the SA matrix, which collectively produced a denser network structure. In terms of chewiness, the highest values were observed for the LBP and CP groups. Since CP and SA both can co-participate in Ca^2+^-mediated gelation, facilitating the formation of a uniform and robust interpenetrating polymer network (IPN), which was responsible for the exceptionally strong chewiness. For the LBP incorporated sample, due to its highly branched configuration, structural reorganization under mechanical stress would facilitate extensive energy dissipation that directly imparted the observed high chewiness value during mastication simulation. Despite the above-mentioned improvements, cohesiveness remained statistically invariant among all groups, indicating the consistent internal bonding integrity regardless of the type of polysaccharides in the capsules. Ta et al. investigated the effects of cross-linking various polysaccharides—κ-carrageenan, tamarind gum, agar and xanthan gum—with calcium alginate to encapsulate *Lactobacillus butyricum* strain 01, and found that compared to calcium alginate alone, gel capsules incorporating these polysaccharides increased the hardness and mechanical strength by approximately 20%, which was attributed to the fact that polysaccharides enhanced the gel network through the formation of physical cross-links and reinforced intermolecular interactions [30].

### 3.4. Rheological Analysis of Probiotic Capsules

**Figure 2:**
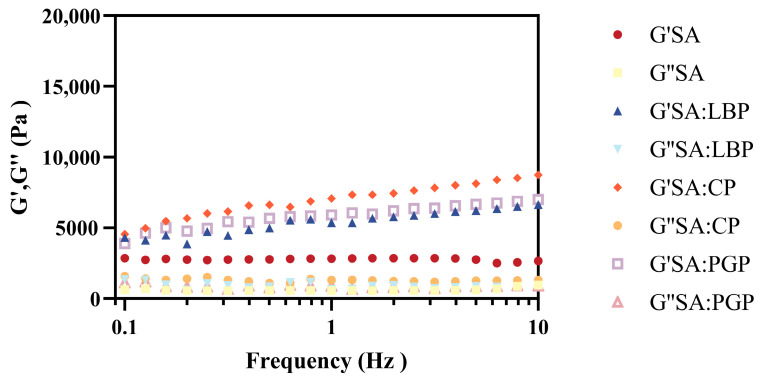
Small-amplitude oscillatory frequency sweeps of different probiotic capsules.

Rheometry characterizes material rheological properties through controlled stress/strain application. As shown in Figure 2, small-amplitude oscillatory shear (SAOS) frequency sweeps were performed to assess viscoelastic evolution and internal structural integrity of probiotic capsules via elastic modulus analysis. As the frequency increased, the storage modulus (G′) of the SA microspheres remained consistent, which can be attributed to the ionically cross-linked network structure that effectively suppressed relaxation behavior. In contrast, the incorporation of polysaccharides resulted in a frequency-dependent increase in G′, which was attributed to the formation of a dynamic viscoelastic network with extended relaxation times. As frequencies swept from 0.0 to 1.0 Hz, the combined system transited into a transient elastic solid, wherein the applied stress was predominantly stored as elastic energy through the stretching of molecular chains and the preservation of cross-links, thereby leading to a pronounced enhancement in G′. The SA: CP group exhibited the highest G′ values at equivalent frequencies, attributable to a more rigid elastic network formed by the synergistic chain entanglement of alginate and citrus pectin, which was further reinforced by Ca^2+^-mediated ionic cross-linking. The loss modulus (G″) was largely frequency-independent across all samples with no significant differences, which can be attributed to the dominant fluid-like viscoelastic response, in which the tendency of G″ was effectively counterbalanced by concurrent shear-thinning behavior, resulting in a frequency-independent loss modulus across the measured range. Besides, for all samples, G′ consistently exceeded G″ without any crossover throughout the tested frequency range, indicating that both the SA matrix and the SA-polysaccharide capsules behave as weak gel systems, satisfying the fundamental gelation criterion (G′ > G″). Furthermore, the addition of polysaccharides exerted a more pronounced effect on the elastic modulus (G′) than on the viscous modulus (G″), underscoring their superior efficacy in enhancing the elasticity of the polysaccharide-SA hybrid gel network compared to the SA group alone [36]. Similar results were found in Zhu et al., who incorporated turmeric polysaccharides into alginate to produce a denser gel network with a higher G′ than pure alginate gels, ascribing this enhancement to improved network cohesion from the introduced intermolecular hydrogen bonding and hydrophobic interactions [37].

### 3.5. Simulated Gastrointestinal Digestion of Probiotic Capsules

**Figure 3:**
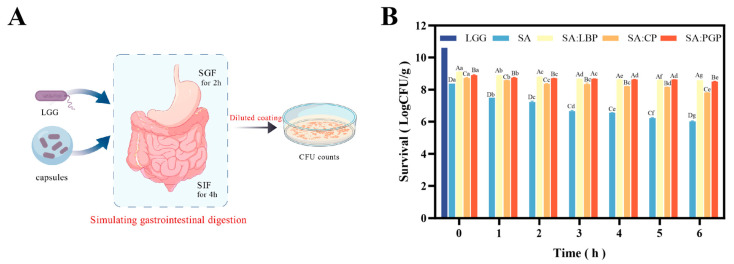
Simulated gastrointestinal digestion of different probiotic capsules. (**A**): Schematic diagram of gastrointestinal digestion; (**B**): Survival rates after simulated gastrointestinal digestion (different letters indicate statistically significant differences between groups (*p* < 0.05)).

To illustrate the simulated gastrointestinal digestion process, a scheme for the probiotic capsules is provided in Figure 3A. The viability profiles of free probiotics (LGG) versus microencapsulated probiotics during 6-h simulated gastrointestinal (GI) digestion are illustrated in Figure 3B. Notably, free probiotics underwent complete inactivation within 1 h of simulated gastric fluid (SGF) exposure, which was attributable to the irreversible cell wall disruption by high H-ion concentration and pepsin proteolysis. Conversely, the viability of LGG encapsulated within SA was significantly enhanced, remaining above 6 log CFU/g even after 6 h of digestion. The protective effect can be attributed to a site-blocking mechanism enabled by the SA-based wall polymers, which reduced direct contact between LGG and digestive enzymes, bile salts, and acidic conditions (H^+^). The carboxylate groups (-COO^−^) on SA underwent protonation under low pH (pH = 2), triggering gelation and the formation of a tightly cross-linked, compact network (denser than that in neutral environments) that collectively maintained the structural integrity and viability of the bacteria [38,39]. Furthermore, the incorporation of various polysaccharides further enhanced the cell survival rates, allowing the microspheres to maintain probiotic viability above 8 log CFU/g after 6 h of simulated gastrointestinal digestion. This improvement can be attributed to two synergistic mechanisms: on one hand, the inherent buffering capacity of the anionic polysaccharides effectively neutralized gastric acid, thereby delayed the initial dissolution of the microspheres; on the other hand, under acidic gastric condition (pH = 2.0), the co-assembly of polysaccharide chains with sodium alginate (SA) promoted the formation of a more complex and densely cross-linked network that effectively filled the interstitial voids within the SA matrix, resulting in a compacted microstructure that provided enhanced physical protection for the encapsulated probiotics [11,40].

The SA: PGP group demonstrated superior probiotic protection during gastrointestinal digestion, as evidenced by the minimal viability loss of only 0.40 log CFU/g, which can be attributed to the fact that PGP can provide additional robust physical barrier against gastric acid and pancreatic enzyme degradation and furthermore, PGP owned high prebiotic activity and can deliver nutritional sustenance that collectively ensured the high probiotic survival throughout digestion [41]. SA: LBP group also conferred notable digestive stability, with only a 0.5 log CFU/g reduction in viability. This well-protection effect stemmed from its structural backbone that is rich in β-1,3 and β-1,6 glycosidic bonds, which were highly resistant to hydrolysis by human digestive enzymes (primarily target α-configurations) and thus remained largely intact during gastrointestinal transit. Similar phenomenon was also found in Zhao et al., who developed a multilayer probiotic microcapsule system using multiphase emulsification technology and found that the structure, with SA encapsulating probiotics in the inner phase, oils in the intermediate phase and a shellac-SA composite forming the outer wall, achieved high encapsulation efficiency and viability retention (>8 log CFU/g), which was attributed to shellac that weakened the porous and pH-sensitive structure of SA, thus suppressed acid penetration and improved the survival of the probiotics during digestion [42].

### 3.6. Storage Stability of Probiotic Capsules

**Figure 4:**
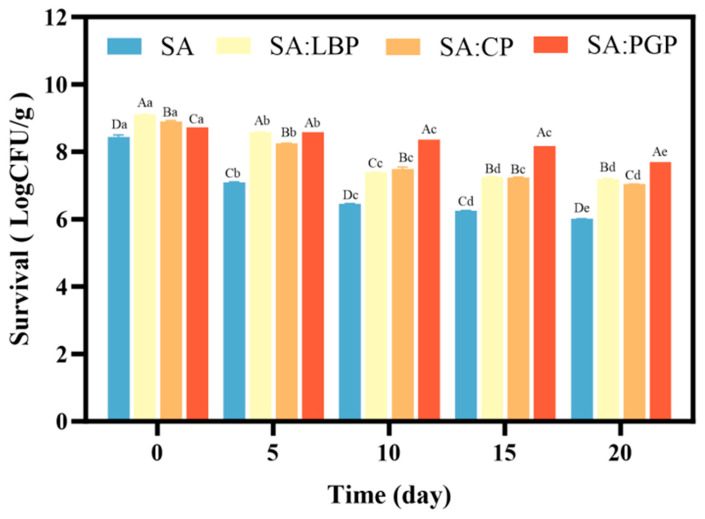
Storage characteristics of probiotic capsules. Different lowercase letters (a–e) denote significant differences between groups at a given time point; different uppercase letters (A–D) denote significant differences over time within a group (*p* < 0.05).

The viability dynamics of three encapsulated probiotics over a 20-day storage period at 4 °C are presented in Figure 4. The Food and Agriculture Organization of the United Nations (FAO)/World Health Organization (WHO) recommends a minimum of 10^6^ CFU/g of live bacteria in probiotic products to ensure efficacy, and herein, during storage, the probiotic viability in all formulations remained stable, consistently exceeding 6 log CFU/g. Probiotic viability in the SA group decreased progressively over the storage period, reaching a final count of 6.01 log CFU/g on day 20, which can be attributed to the accumulation of oxidative damage: sustained elevated metabolic activity increased reactive oxygen species (ROS) levels while reducing key antioxidant enzyme activity (e.g., SOD and CAT), the consequent oxidation of cellular components, including lipids and proteins, systematically impaired membrane and functional integrity, resulting in progressive viability loss [43]. The incorporation of polysaccharides significantly enhanced probiotic viabilities during storage compared to that in SA group, which can attribute to the synergistic physical and metabolic effects: on one hand, polysaccharides integration yielded a more continuous capsule surface with reduced porosity and thus effectively shielded probiotics from environmental stresses; on the other hand, the gradual degradation of these prebiotic polysaccharides can potentially supply sustained nutrients, moderate bacterial metabolic activities, and thereby collectively enhanced storage stability [44,45]. Among these groups, SA: PGP exhibited the highest storage stability, which still maintained 7.7 Log CFU/g of viable cells at the end of storage time and was thus selected to apply in yogurt below. The superior performance can be attributed to the significant antioxidant capacity of PGP that effectively scavenges environmental free radicals such as ROS and provides further protection through its abundant carboxyl and hydroxyl groups, which have the ability to chelate redox-active metal ions [46,47,48]. Similar results can be found in Yao et al., who prepared probiotic microcapsule via spray drying, *Lactobacillus plantarum* 550 encapsulated in soy protein isolate (SPI) and PGP, and found that the incorporation of PGP exhibited significantly enhanced probiotic viability as compared to that in control group following 8-week storage; meanwhile, the cell viabilities demonstrated a positive correlation to the PGP concentration gradient [41].

### 3.7. Application of Probiotic Capsules in Yogurt

**Figure 5:**
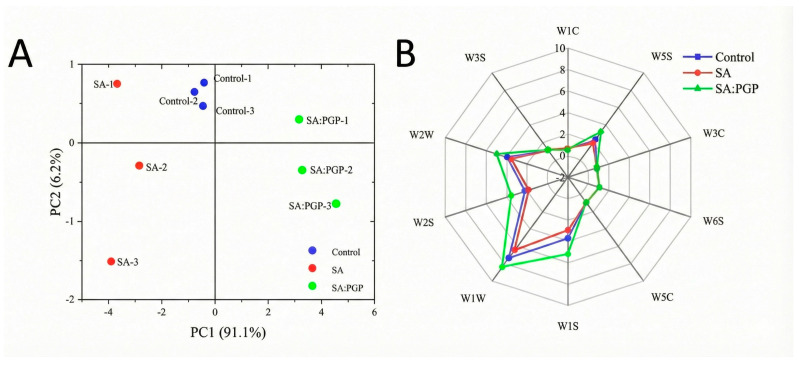
Flavor analysis of different yogurts. (**A**): PCA diagram; (**B**): Electronic nasal odor radar chart.

Electronic nose (E-nose) technology rapidly identifies volatile compounds by simulating human olfaction with sensor arrays, and principal component analysis (PCA) was applied to further evaluate odor profile alterations induced by probiotic capsule incorporation. As depicted in Figure 5A, PCA accounted for 97.3% of total variance (PC1 + PC2), validating model feasibility and demonstrating clear odor discrimination among yogurts. The control, SA, and SA: PGP samples clustered in distinct spatial zones, confirming the significant differences in their global volatile signatures. Complementary flavor fingerprint radar analysis (Figure 5B) revealed differential sensor responses to key volatiles, specifically: W1W (sulfides), W2W (aromatics and organ sulfides), and W1S (alkanes), W5S (nitrogen oxides). Notably, the SA: PGP group exhibited significantly higher sensitivity to W1W, W2W, and W1S sensors compared to both the control and SA groups, suggesting an enhanced release of sulfur-containing compounds and alkanes in SA: PGP yogurts. This flavor enhancement was primarily mediated by PGP, which functioned as a prebiotic by sustaining the metabolic activity of LGG and thereby supported amino acid fermentation for the production of key volatiles such as sulfides and alkanes. Conversely, the control group, lacking nutritional support, exhibited accelerated nutrient depletion, which suppressed microbial metabolism and limited the synthesis of complex volatile compounds. Similar observations were reported by Fan et al., who investigated the yogurt supplemented with dual-strain probiotics and wolfberry-derived dietary fiber (WDF), and found that polysaccharide fiber incorporation effectively modulated volatile compound profiles in fermented dairy systems [49].

**Figure 6:**
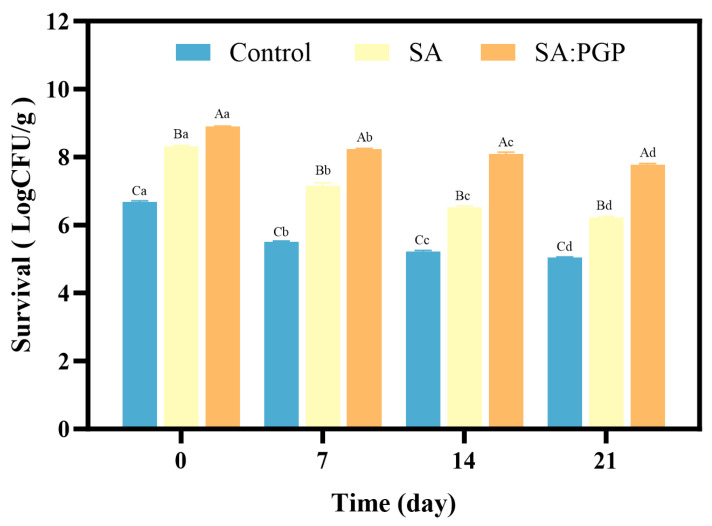
Storage characteristics of different yogurts. Different lowercase letters (a–d) denote significant differences between groups at a given time point; different uppercase letters (A–C) denote significant differences over time within a group (*p* < 0.05).

The microbial growth kinetics were used to evaluate the yogurt stability under extended storage conditions (Figure 6). The control group demonstrated the lowest survival rate, with a final count of only 5.05 log CFU/g remaining by day 21. The decline in viable probiotic counts correlated with the loss of cell membrane integrity, driven by phospholipid peroxidation and accumulation of saturated fatty acids [50]. The SA group exhibited a rapid reduction in viability during the initial storage phase (0–7 days), a process attributable to nutrient depletion-induced metabolic stress arising from a starvation state prompted by the exhaustion of bioavailable substrates under hermetic conditions, which consequently diminished the cell stress resistance capacity. In contrast, the incorporation of PGP significantly enhanced the storage stability of probiotic yogurt, which can attribute to, on one hand, PGP interacted tightly with sodium alginate to form a dense continuous and imporous capsule surface that physically shielded probiotics from environmental stressors; on the other hand, PGP owned potential prebiotic activity that can modulate bacterial metabolism and mitigate oxidative damage, thereby collectively ensured higher probiotic viability throughout storage. Similar observations were reported by Yang et al., who fermented goat yogurt using four probiotic strains with or without encapsulation, among which cells with microencapsulation demonstrated significantly high potentials in enhancing the survivability of *Bifidobacterium* by mitigating the adverse conditions inherent in goat yogurt [51].

## 4. Conclusions

This study demonstrated that the incorporation of three plant-derived polysaccharides, lycium barbarum polysaccharides (LBP), peach gum polysaccharide (PGP), and citrus pectin (CP), with alginate (SA) to co-encapsulate probiotics can significantly improve the encapsulation efficiency, structural regularities, in vitro gastrointestinal digestion, and storage stability of cells. Among these, the SA: PGP group showed the superior protection effects due to its densest gel structure and prebiotic potential. The formula developed in this study offers promise for widespread use in probiotics, and further investigation and optimization of this formula can lead to commercial products.

## Figures and Tables

**Table 1:** Encapsulation efficiencies of different probiotic capsules.

Sample	SA	SA: LBP	SA: CP	SA: PGP
1:2 1:1 2:1	79.27 ± 0.07 ^D^	84.26 ± 0.13 ^Ac^ 85.04 ± 0.05 ^Ab^ 86.07 ± 0.11 ^Aa^	82.40 ± 0.06 ^Cc^ 83.09 ± 0.04 ^Cb^ 83.90 ± 0.10 ^Ca^	83.59 ± 0.07 ^Bb^ 83.63 ± 0.19 ^Bb^ 84.14 ± 0.06 ^Ba^

Note: Columns with different superscripts (a, b, c) are significantly different (*p* < 0.05). Lines with different superscripts (A, B, C, D) are significantly different (*p* < 0.05).

**Table 2:** Particle size analysis of different probiotic capsules.

Sample	Size (mm)
SA	2.14 ± 0.01 ^c^
SA: LBP	2.33 ± 0.04 ^a^
SA: CP	2.25 ± 0.03 ^b^
SA: PGP	2.37 ± 0.03 ^a^

Note: Columns with different superscripts (a, b, c) are significantly different (*p* < 0.05).

**Table 3:** Texture analysis of different probiotic capsules.

Sample	Hardness	Springiness	Cohesiveness	Chewiness	Resilience
SA	50.90 ± 1.43 ^c^	0.62 ± 0.02 ^a^	0.71 ± 0.01 ^a^	23.28 ± 0.23 ^c^	0.22 ± 0.04 ^b^
SA: LBP	55.82 ± 0.16 ^b^	0.58 ± 0.01 ^b^	0.71 ± 0.01 ^a^	25.57 ± 0.60 ^a^	0.23 ± 0.06 ^ab^
SA: CP	56.64 ± 0.20 ^b^	0.59 ± 0.01 ^b^	0.71 ± 0.09 ^a^	25.17 ± 0.14 ^a^	0.29 ± 0.01 ^ab^
SA: PGP	71.50 ± 0.12 ^a^	0.64 ± 0.01 ^a^	0.71 ± 0.03 ^a^	24.47 ± 0.21 ^b^	0.30 ± 0.02 ^a^

Note: Columns with different superscripts (a, b, c) are significantly different (*p* < 0.05).

## Data Availability

The original contributions presented in this study are included in the article. Further inquiries can be directed to the corresponding authors.
